# Identifying the time scale of synchronous movement: a study on tropical snakes

**DOI:** 10.1186/s40462-015-0038-5

**Published:** 2015-05-04

**Authors:** Tom Lindström, Benjamin L Phillips, Gregory P Brown, Richard Shine

**Affiliations:** Department of Physics, Biology and Chemistry, Linköping University, 58183 Linköping, Sweden; School of Biosciences, University of Melbourne, Melbourne, VIC 3010 Australia; School of Biological Sciences, University of Sydney, Sydney, NSW 2006 Australia

**Keywords:** Periodogram, Hierarchical Bayes, Relocation data, Elapidae, Ectotherms

## Abstract

**Background:**

Individual movement is critical to organismal fitness and also influences broader population processes such as demographic stochasticity and gene flow. Climatic change and habitat fragmentation render the drivers of individual movement especially critical to understand. Rates of movement of free-ranging animals through the landscape are influenced both by intrinsic attributes of an organism (e.g., size, body condition, age), and by external forces (e.g., weather, predation risk). Statistical modelling can clarify the relative importance of those processes, because externally-imposed pressures should generate synchronous displacements among individuals within a population, whereas intrinsic factors should generate consistency through time within each individual. External and intrinsic factors may vary in importance at different time scales.

**Results:**

In this study we focused on daily displacement of an ambush-foraging snake from tropical Australia (the Northern Death Adder *Acanthophis praelongus*), based on a radiotelemetric study. We used a mixture of spectral representation and Bayesian inference to study synchrony in snake displacement by phase shift analysis. We further studied autocorrelation in fluctuations of displacement distances as “one over f noise”. Displacement distances were positively autocorrelated with all considered noise colour parameters estimated as >0. We show how the methodology can reveal time scales of particular interest for synchrony and found that for the analysed data, synchrony was only present at time scales above approximately three weeks.

**Conclusion:**

We conclude that the spectral representation combined with Bayesian inference is a promising approach for analysis of movement data. Applying the framework to telemetry data of *A. praelongus*, we were able to identify a cut-off time scale above which we found support for synchrony, thus revealing a time scale where global external drivers have a larger impact on the movement behaviour. Our results suggest that for the considered study period, movement at shorter time scales was primarily driven by factors at the individual level; daily fluctuations in weather conditions had little effect on snake movement.

**Electronic supplementary material:**

The online version of this article (doi:10.1186/s40462-015-0038-5) contains supplementary material, which is available to authorized users.

## Background

One of the most basic questions that can be asked in behavioural ecology is “when, and why, do animals move?” Movement may be critical for an individual’s fitness (influencing its ability to forage and find mates), and may also contribute to its inclusive fitness (via dispersal and concomitant reduction in kin competition). However, movement is also costly (in energy and predation risk) and is constrained by external conditions (e.g., cold temperatures constrain movement in ectotherms). The fundamental questions of when and why an animal moves is difficult to resolve because multiple competing factors influence an individual’s decision of when and how far to move.

Typically, when analysing patterns of movement (or surrogates such as encounter rates or “trapability”) over time, researchers treat movement rates in each time period as independent, and then correlate movement rates with environmental or state variables measured within each of those time periods. This approach suffers from two major drawbacks. First, movement decisions may be based on some unmeasured (and/or unsuspected) aspect of the environment. In practice, this leads to testing for correlation with a large number of potential explanatory variables, inflating type I error rates (e.g., [1]). Second, movement between time periods is unlikely to ever be truly independent, and the scale at which this non-independence plays out may also not be discrete. That is, an individual’s movement today may be influenced by what it did yesterday (and indeed, throughout its earlier life). Attempts to test for correlations with explanatory variables may yield spurious results if the statistical method employed doesn’t correctly account for the autocorrelation [2]. One means of overcoming the risk of misinterpreting autocorrelated data is the application of statistical tests that allow explicit modelling of autoregressive (AR) errors (e.g., time series analyses [3,4]). Here, we take an alternative approach and simply ask whether movement of individuals is synchronous. Synchronous movement would indicate that activity is driven or limited by external environmental factors that affect the whole population, possibly in combination with internal, rhythmic synchronization. A lack of synchrony, however, would imply that neither environmental nor rhythmic synchronization is occurring and, therefore, that any search for global factors to explain movement is pointless.

The importance of factors at the individual level vs global external factors may further depend on the time scale that is considered. At larger time scales, seasonal patterns in food availability may cause synchronous movement [5,6]. For animals with clear migratory behaviour, such as Monarch butterflies [7] or wildebeest [8], synchronization may be obvious at the seasonal scale. More subtle external synchronization may be present within a season, but the time scale at which this occurs may be less evident. Resolving this timescale can clarify which external factors affect movement pattern, and consequently what temporal resolution should be considered when testing for explanatory variables. Further, the approach we use here (spectral analysis) clarifies the autocorrelation structure of the data across multiple time scales, thus identifying time scales of particular importance in the movement pattern.

In this study, we investigate whether the movement of a large tropical ectotherm, the northern death adder (*Acanthophis praelongus*) is influenced by global (climatic) variables, or by factors working at the individual level; and how this relationship depends on the time scale considered. The northern death adder is a relatively large (up to 500 g) terrestrial ambush-foraging elapid snake. As with all snakes, we might expect movement of death adders to be sensitive to weather conditions, especially temperature. In temperate climates, most days of the year may be too cold for snakes to move [9,10]. In tropical environments however, thermoregulation may cause snakes to stay out of the sun to avoid overheating [11,12]. Snakes also alter their movement behaviour in response to other climatic factors such as humidity [13-15], wind speed [14] and precipitation [16].

Snake movement is also affected by trophic interactions. Predation risk is generally higher when the individual is active [17] and the individual may alter its movement behaviour in response to its perception of predation risk. Prey availability may also influence snake movement in several ways. Individuals are expected to perform area restricted search if they perceive a high abundance of prey, but to shift location if rates of prey encounter are low [5,18]. Subsequent to catching prey, a snake is further expected to reduce movement rates in order to digest the prey. At a shorter time scale, these trophic interactions are likely to influence movement mainly at the individual level. However, large-scale fluctuations in prey availability may cause synchronous (seasonal) movement of many individuals within a snake population [6].

Our aim here is to investigate if the movement of *A. praelongus* is driven by some global, external factors (such as weather conditions) that influence the displacement of all snakes. Hastings [19] pointed out the value of using phase shift analysis in order to study synchrony in ecological time series and we here implement a Bayesian approach for hypothesis testing based on Deviance Information Criterion (DIC) [20]. In order to provide insight into the relevant timescales of *A. praelongus* movement ecology, we also study the autocorrelation patterns of its movement. By spectral representation and (hierarchical) Bayesian modelling, we analyse fluctuations in displacement distances as “one over f noise” [21].

## Methods

### Study site and data collection

Here we use a subset of movement data that was collected during a study designed to examine survival rates in free-ranging death adders following the arrival of an invasive species [22]. Snakes were tracked at Fogg Dam, in the Northern Territory, Australia. In total, 54 individuals were included in the study, but most of these were only tracked for shorter periods, leaving them unsuitable for the purpose of identifying synchrony at different time scales. Our analysis also requires data to be continuous (i.e., no breaks in the individual time series), and for multiple individuals to be measured contemporaneously. Of the total data collected for the work in Phillips et al. [22], we therefore chose a 64-day period (beginning 3 February 2006, in the “Wet Season”) across which six adders were relocated every day via radiotelemetry (for detailed methods, see Phillips et al. [22]). All individuals were males, which is likely to be a result of sex-biased mortality in response to the cane toad invasion [23]. Although the restriction of our analysis to males prevents us from investigating differences between males and females, it avoids problems with possible asynchronous behaviour induced by sexual dimorphism. Adders were tracked on the Adelaide River Floodplain in Australia’s Northern Territory adjacent to Beatrice Hill (all adders were released at 12.640141°S, 131.315548°E).

Movement distances of death adders are positively skewed, with higher probability of shorter movements. In the spectral analysis below, we assume frequency components to be independent and at moderate levels of autocorrelation we should expect approximately zero skewness [24]. In order to reduce the skewness of displacement distances we performed a power transform of the data by ${X}_{t,n}={D}_{t,n}^c$, where *D*_*t,n*_ is the displacement distance of individual *n* at time *t* and *c* is calculated by minimizing ${\displaystyle \sum_{n=1}^N}\left|\mathrm{Skewness}\left({D}_{\cdot, n}^c\right)\right|$. Here, *D*_⋅,*n*_ refers to all displacement distances of individual *n* and *N* is the number of individuals (i.e. *N* = 6). We estimated c = 0.17 and performed the analysis of synchrony and autocorrelation on *X*_*t*,*n*_.

### Synchrony of movement

We investigated whether adder movement was synchronous and identified the time scale on which such synchrony was present. We did this by extending a method presented by Lindström et al. [25], based on a combination of phase shift analysis and Bayesian inference. A time series of length *L* has *L*/2 wave components, however when *L* is an even number (as it is in this data set), the phase of the highest frequency is either 0 or _*π*_ and is here excluded from the analysis of phase and we define *l* as the *l* = *L*/2-1 = 31 We define **θ** as a matrix of dimensions *l* × *N*, where *θ*_*f*,*n*_ is the phase of frequency *f* for the time series of individual *n*.

The basic outline of the analysis is to determine if phases of each considered frequency are better described by some circular distribution, here a Wrapped Cauchy Distribution (WCD), centred at a mean phase *μ*_*f*_, or by a uniform circular distribution, Uniform (0, 2*π*). The WCD is given by

1.1 $$ P\left({\theta}_{f,n}\Big|{\mu}_f,{\rho}_f\right)=\frac{1}{2\pi}\frac{1-{\rho}_f^2}{1 + {\rho}_f^2-2{\rho}_f \cos \left({\theta}_{f,n}-{\mu}_f\right)} $$

where *ρ*_*f*_ is the mean cosine of the distribution of phases for frequency *f* and describes the level of phase synchrony for that frequency, with *ρ*_*f*_ = 0 indicating no synchrony and *ρ*_*f*_ = 1 indicating perfect synchrony*.* We want to find the frequency *φ* where the phase data is better described as synchronous for 0 < *f* ≤ *φ* and random for frequencies *φ* < *f* ≤ *l*, where *l* is the total number of frequencies. If we can locate such cutoff, we can conclude that synchrony is present for time scales modeled by *f* for 0 < *f* ≤ *φ* but not for *φ* < *f* ≤ *l*. The likelihood is written

1.2 $$ P\left(\boldsymbol{\uptheta} \Big|\boldsymbol{\upmu}, \boldsymbol{\uprho} \right)=\left\{{\displaystyle \prod_{f=1}^{\varphi }{\displaystyle \prod_{n=1}^NP\left({\theta}_{f,n}\Big|{\mu}_f,{\rho}_f\right)}}\right\}{\left(2\pi \right)}^{-N\left(l-\varphi \right)} $$

where **μ** and **ρ** are vectors of length *l*, containing the frequency specific parameters *μ*_*f*_ and *ρ*_*f*_, respectively, for *f* = 1,2…,*l*.

A caveat of this study is the number of individuals available. With *N* = 6, only six data points are available to assess the presence or absence of synchrony for each frequency. One way to circumvent this problem would be to construct a model where *ρ*_*1*_ = *ρ*_*2*_ = … *ρ*_*φ*_. This however would assume identical phase synchrony for all frequencies 0 < *f* ≤ *φ*, which seems too crude an assumption. However, we may expect that synchrony is not entirely independent and by constructing a hierarchical Bayesian model, we allow for “borrowing strength” [26] between frequencies. The Bayesian model then has the form

1.3 $$ P\left(\boldsymbol{\upmu}, \boldsymbol{\uprho}, M,\nu \Big|\boldsymbol{\uptheta} \right)\propto P\left(\boldsymbol{\uptheta} \Big|\boldsymbol{\upmu}, \boldsymbol{\uprho} \right)P\left(\boldsymbol{\uprho} \Big|M,\nu \right)P\left(\boldsymbol{\upmu} \right)P(M)P\left(\nu \right), $$

where *P*(**ρ**|*M*, *ν*) is the hierarchical distribution for **ρ**. This is here chosen to be a beta distribution defined by its mode *M* and shape *ν* = *α + β*, where *α* and *β* are the two shape parameters of the standard parameterization of the beta distribution. Further, *P* (**μ**), *P* (*M*) and *P* (*ν*) indicate (hyper) prior distributions.

We use Deviance Information Criterion (DIC) for model selection, $\mathrm{D}\mathrm{I}\mathrm{C}=2{p}_D+D\left(\overline{\boldsymbol{\upxi}}\right)$, where *p*_*D*_ is the effective number of parameters and $D\left(\overline{\boldsymbol{\upxi}}\right)$ is the deviance of the expected value of parameters, $$ \overline{\boldsymbol{\upxi}}=\left[\begin{array}{c}\hfill \overline{\boldsymbol{\upmu}}\hfill \\ {}\hfill \overline{\boldsymbol{\uprho}}\hfill \end{array}\right] $$. Here, $\overline{\boldsymbol{\upmu}}$ and $\overline{\boldsymbol{\uprho}}$ are vectors of length *l*, containing the arithmetic and circular mean, respectively, of the posterior estimates of **μ** and **ρ**, respectively.

### The colour of movement

While synchrony is the main focus of our study, the autocorrelation pattern provides additional information about processes influencing movement. Recent studies [27,28] have demonstrated the power of spectral methods to extract information from animal relocation data. The relationship between frequency and amplitude can be used to analyse autocorrelation patterns. This is conveniently expressed in the periodogram, which contains information about how the variability in the data is distributed over different frequencies. Positively autocorrelated time series are commonly denoted “red” because (like red light) they are dominated by sine wave components of low frequencies. For the same reason, negatively autocorrelated time series are dominated by high frequencies and denoted “blue”, and time series with no relation between frequency and amplitude are considered “white”. The calculations for obtaining the periodogram and phases of a time series are based on Fast Fourier Transform (FFT) and may be found in [25].

Within ecological studies of environmental fluctuations, the analysis of time series colour is commonly performed by what is known as “one over f noise” [21]. This has proven to be a good model for both temporal [29] and spatial [30] considerations of ecological systems and assumes that Periodogram(*f*) ∝ *f*^− *γ*^, where *f* is frequency and the colour parameter *γ* is negative for blue noise, positive for red, or zero for white. This model is linear when considered on log-log axes and commonly *γ* is calculated as the slope of this linear fit of log (*f*) vs log (periodogram). This however only provides a single point estimate and does not include parameter uncertainty. Here we instead use hierarchical Bayesian analysis and thereby obtain posterior probabilities of parameters at both the individual and population level. As such, we acknowledge the uncertainty of parameters at the individual level when estimating the population level parameters.

We define **ψ** as a matrix of dimensions *l* × *N* where the element *ψ*_*f*,*n*_ s the periodogram ordinate of individual *n* and frequency *f*. We follow the Whittle approximation [31] for modelling of periodogram ordinates and model the probability of the periodogram of frequency *f* of individual *n* as $\mathrm{E}\mathrm{x}\mathrm{p}\left({\psi}_{f,n}\Big|{Y}_{f,n}^{-1}\right)$ where ${Y}_{f,n}={a}_n{f}^{-{\gamma}_n}$. Here, *γ*_*n*_ is the colour parameter for individual *n* and *a*_*n*_ is an individual specific nuisance parameter related to the overall magnitude of the fluctuations. Defining ***a*** as *a*_1_, *a*_2_…*a*_N_, and *γ*_1_, *γ*_2_… *γ*_N_, the full hierarchical Bayesian model for the periodogram is written

1.4 $$ P\left(\boldsymbol{a},\boldsymbol{\gamma}, {\boldsymbol{\xi}}_a,{\boldsymbol{\xi}}_{\gamma}\Big|\boldsymbol{\psi} \right)\propto {\displaystyle \prod_{n=1}^N}\left[\left[{\displaystyle \prod_{f=1}^m}\mathrm{E}\mathrm{x}\mathrm{p}\left({\psi}_{f,n}\Big|{Y}_{f,n}^{-1}\right)\right]P\left({a}_n\Big|{\boldsymbol{\xi}}_a\right)P\left({\gamma}_n\Big|{\boldsymbol{\xi}}_{\gamma}\right)\right]P\left({\boldsymbol{\xi}}_a\right)P\left({\boldsymbol{\xi}}_{\gamma}\right), $$

where *P*(*a*_*n*_|***ξ***_*a*_) and *P*(*γ*_*n*_|***ξ***_*γ*_) are hierarchical priors with hyper parameters ***ξ***_***a***_ and ***ξ***_***γ***_, respectively, and hyper priors *P*(***ξ***_*a*_) and *P*(***ξ***_*γ*_), respectively. We define ${\boldsymbol{\xi}}_{\gamma}\equiv \left(\overline{\gamma},{\sigma}_{\gamma}\right)$ and model ${\gamma}_n\sim \mathrm{Normal}\left(\overline{\gamma},{\sigma}_{\gamma}\right)$. Because *a*_*n*_ is inherently positive, we define ***ξ***_*a*_≡(*ā*_log_, *σ*_*a* log_) and model log(*a*_*n*_) ~ Normal(*ā*_log_, *σ*_*a* log_). The main hierarchical parameter of interest in this study is $\overline{\gamma}$, which may be interpreted as a population level measure of autocorrelation and is estimated such that uncertainty at the individual level is incorporated. Elicitation of hyper priors *P*(***ξ***_*a*_) and *P*(***ξ***_*γ*_) are treated in Prior elicitation(below). Parameters are estimated by Markov Chain Monte Carlo (MCMC) as described by [25], and computation was performed in MATLAB (The MathWorks, Inc., Natick, Massachusetts, United States).

In order to validate the choice of likelihood function (i.e. 1/f model with independent, exponentially distributed ordinates) we also perform a residual test. This is essential because the Whittle approximation may be violated when considering highly autocorrelated time series. For each individual, we repeatedly generate realizations of ${Y}_{f,n}={a}_n{f}^{-{\gamma}_n}$ by random, joint draws of *a*_*n*_ and *γ*_*n*_ from the posterior distribution (as given by the MCMC) and calculate *Z*_*f*,*n*_ = *ψ*_*f*,*n*_/*Y*_*f*,*n*_. We test for autocorrelation by calculating the Pearson correlation for autocorrelation of lag one, five and ten and for each individual we analyse the autocorrelation by the proportion of realizations that generated a significant (by p < 0.05 limit) autocorrelation. Similarly, we test for the assumption of exponentially distributed ordinates by the proportion of realizations that tested significant by a Kolmogorov-Smirnov test. We consider tests significant if more than 50% of the realizations yields p < 0.05.

### Prior elicitation

Our aim here is to use vague priors, hence making inference mainly based on the data. For synchrony parameters, this is promoted by the alternative parameterisation of the beta distribution used for *P*(**ρ**|*M*, *ν*). For the mode parameter *M*, a vague prior can be defined as *P*(*M*) = Uniform(0, 1)*.* It is difficult to have informative a priori beliefs about the shape parameter *ν*, and we therefore define a vague hyperprior. For this purpose we want to allow the possibility of both large and small values, corresponding to small and large difference among frequency specific synchronies **ρ**, respectively. For this purpose, we specify *P* (*ν*) as a gamma distribution with 95% of its density between five (large difference) and 200 (small difference). We further specify *P*(**μ**) = Uniform(−*π*, *π*).

To ensure that our results are insensitive to the choice of prior, we perform a sensitivity analysis. First we investigate the sensitivity to the choice of *P* (*ν*) and implement two alternative priors: one that that has 95% density between five and ten (i.e. informative with high probability of low values), and one that has 95% density between 100 and 200 (i.e. informative with high probability of high values).

We also investigate the effect of *P* (*M*) by using two alternative distributions: Beta (0.5,0.5) and Beta (5,5). Compared to the uniform distribution, these distributions have larger and smaller variance, respectively.

For the parameters used in the analysis of the autocorrelation pattern, we implement conjugate priors $\overline{\gamma}\sim \mathrm{Normal}\left(\widehat{\gamma},{\widehat{\tau}}_{\overline{\gamma}}^2\right)$ and ${\sigma}_{\gamma}^2\sim \mathrm{I}\mathrm{n}\mathrm{v}\hbox{-} {\upchi}^2\left({\nu}_0,{\sigma}_0^2\right)$, respectively, where Inv ‐ χ^2^ is the scaled inverse *χ*^2^ distribution. We set $\widehat{\gamma}=0$ and ${\widehat{\tau}}_{\overline{\gamma}}^2=4$, hence specifying a prior for $\overline{\gamma}$ that has 95% of the density between -4 and 4. This prior belief includes both highly negative and positive values of population level autocorrelation $\overline{\gamma}$. Following suggestions from Gelman et al. [26], the hyperparameters ν_0_ and ${\sigma}_0^2$ were implicitly given from our prior beliefs of about ${\sigma}_{\gamma}^2$ in terms of the most likely value (i.e. the mode) and some upper percentile. We use mode = 0.0625 and an upper 95% percentile of one. The mode follows from the assumption that the standard deviation of the distribution of ***γ*** around $\overline{\gamma}$ is most likely 0.25 (i.e. approximately 95% of individual *γ*_*n*_ approximately within $\overline{\gamma}\pm 0.5$) and the upper percentile follows from the assumptions that we are 95% sure that *σ*_*γ*_ < 1 (i.e. 95% of individual *γ*_*n*_ approximately within $\overline{\gamma}\pm 2$).

A priori beliefs about *ā*_log_ and *σ*_*a* log_ are intricate because ***a*** not only relate to the magnitude of the fluctuations of displacement distances, but also depend on *γ*. We therefore use an improper but flat prior with *P*(*ā*_log_) ∝ 1 and $P\left({\sigma}_{a \log}^2\right)\propto 1$.

## Results

**Figure 1:**
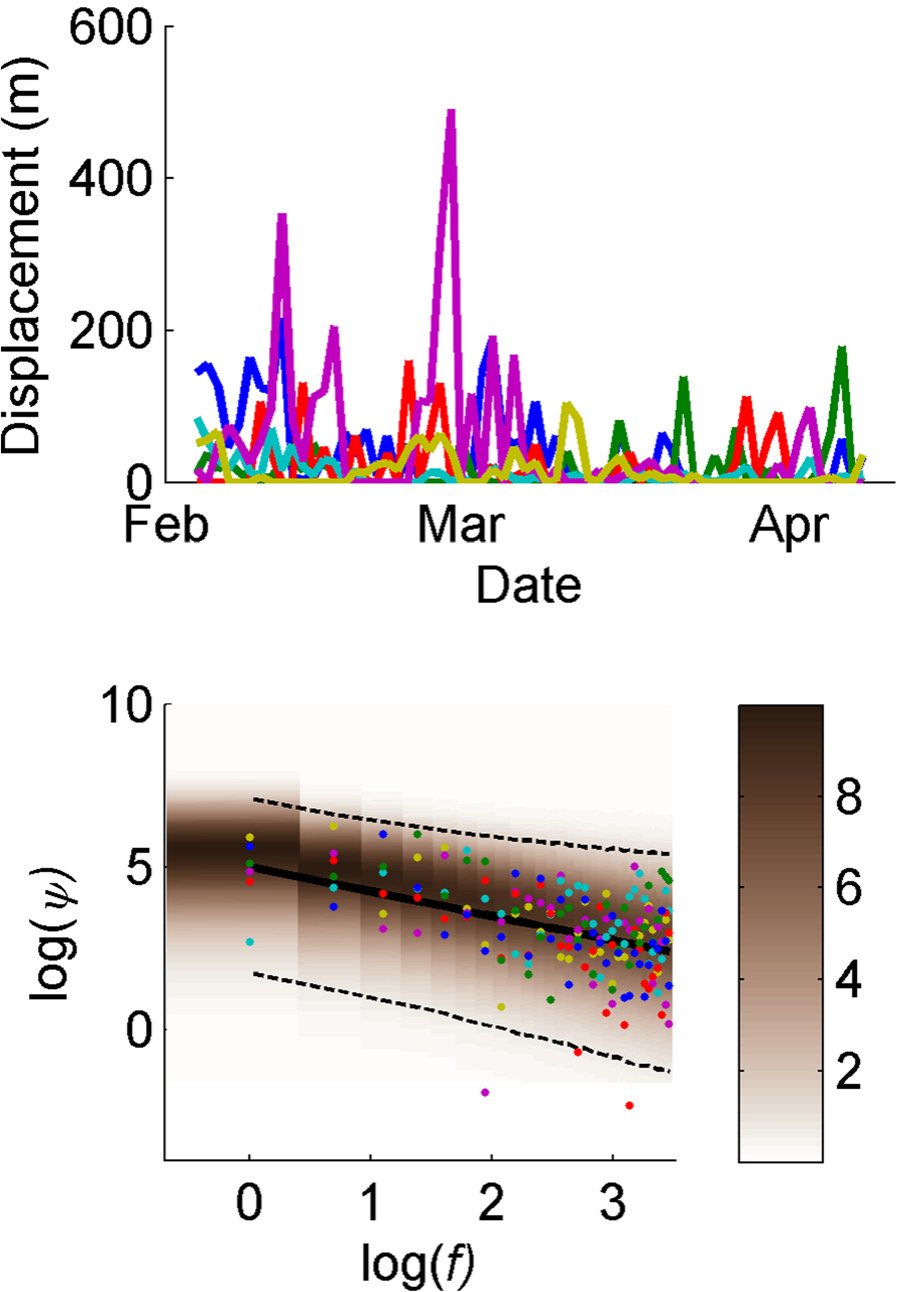
Top panel: individual daily displacement of *Acanthophis praelongus*. Lower panel: Observed (dots with one colour per snake corresponding to legend in top panel) and posterior predictive distribution (shaded with density as indicated by colourbar and mean and 95% central density indicated by solid and dotted lines, respectively) of periodogram (*ψ*).

Table 1 provides some descriptive statistics and Figure 1 (top panel) shows the daily displacement of the different individuals. The full data is available in Additional file 1: Table S1.

**Table 1:** **Descriptive statistics of the six** ***Acanthophis praelongus*** **individuals included in the study**

**Individual**	**1**	**2**	**3**	**4**	**5**	**6**
Mean distance (m)	39	17	20	12	49	15
Standard deviation of distances (m)	55	32	38	17	92	24
Cumulative distance (m)	2487	1073	1261	749	3123	975
Maximum distance (m)	216	179	161	84	490	105
Start position (easting)	751346	751474	751470	751524	751784	751375
Start position (northing)	861438	861494	861700	861580	861572	861752
End position (easting)	751351	751645	751400	751582	751321	751610
End position (northing)	861778	861438	861930	861760	861769	861706

Distance measures refer to daily displacement and positions to UTM WGS84 datum coordinates.

### Synchrony

**Figure 2:**
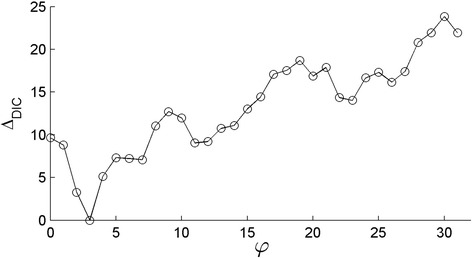
Difference in DIC (Δ_DIC_) for models describing the data as synchronous for frequencies *f* ≤ *φ*. Each frequency corresponds to a time scale of *L*/*f* and the lowest value is found for *φ* = 3, corresponding to 21 days.

Figure 2 plots Δ_DIC_, defined as the difference between model DIC and DIC_min_, where the latter is the DIC of the model with the lowest score. This was found for *φ* = 3, which corresponds to a time scale of 21 days (given by 64/3). Spiegelhalter et al. [20] suggest that a difference in DIC of more than three constitute a model with considerably less support than the preferred model. Figure 2 shows that the jump between *φ* = 3 and *φ* = 4 is greater than this cutoff, suggesting that the best model describes the data as being synchronous only for frequencies equal to and below this cutoff. Consequently, we conclude that for these data, movement is synchronous at time scales above three weeks but not at shorter time scales, indicating that factors that cause synchronization only act at these larger time scales, whereas daily fluctuations of movement are regulated by factors at the individual level (such as internal drivers).

The sensitivity analysis showed that the results were insensitive to the choice of hyperprior. The lowest DIC was consistently found for *φ* = 3, with the model with *φ* = 4 having considerably less support than the preferred model (Δ_DIC_ within the range three to seven). Figures that correspond to Figure 2 but with the alternative priors are presented as an electronic supplement Additional file 2: Figure S1 to this paper.

### Colour

The colour parameter estimates of snake displacements are shown in Table 2. Marginal posterior estimates for both individual parameters (***γ***) and population parameters ($\overline{\gamma}$) were all clearly positive, indicating that snake movement is positively autocorrelated (that is, a snake that moved a long way yesterday, is also likely to move a long way today). In the residual analysis, individuals three and five showed significant autocorrelation at lag one, yet at lag five and ten, only individual three was significant. The Kolmogorov-Smirnov test showed no significant deviations from exponential distribution. The model prediction (as by posterior predictive distribution) and observed periodograms are presented in Figure 1 (lower panel) that aims to clarify time scales where the model predictions deviate from the observed data. Although individual snakes may deviate at some time scales; generally Figure 1 illustrates a good fit between the log-linear assumptions of the 1/f-noise model and the observed periodograms of snake movements.

**Table 2:** **Mean estimates of the marginal posterior distribution of autocorrelation parameter** ***γ*** **for all six considered snakes and population level parameter** ***γ***

**Parameter**	***γ***_**1**_	***γ***_**2**_	***γ***_**3**_	***γ***_**4**_	***γ***_**5**_	***γ***_**6**_	$\overline{\gamma}$
Estimate	0.82 [0.48, 1.1]	0.72 [0.34, 0.97]	0.60 [0.16, 0.85]	0.91 [0.52, 1.2]	0.60 [0.25, 0.84]	0.89 [0.53, 1.1]	0.75 [0.32, 1.0]

Brackets indicate 95% central credibility interval.

## Discussion

Our analysis of snake movement consists of two parts: a phase shift analysis, which analysed between-individual synchrony, and a colour analysis which estimated autocorrelation in daily displacements of individuals. The phase shift analysis demonstrated that synchrony in displacement distances of snakes was only present at time scales above three weeks. Our analysis therefore suggests that among this sample of snakes, external, global factors were only likely to be important at these larger time scales. At shorter time scales, the movement behaviour of these radio-tracked snakes was primarily driven by factors that varied among individuals. If displacement distances are interpreted as a proxy for activity, daily fluctuations in global conditions (such as daily temperature, humidity, or number of sunspots) did not determine activity of the snakes in this study: at shorter time scales, the movement of one individual says very little about the movement of others. This suggests that in order to find environmental drivers of snake movement, we need to look for environmental variation acting at longer timescales (greater than 21 days). We cannot identify the underlying cause for the tendency towards synchrony above 21 days, but it is likely that seasonal variation in climatic variables and/or food availability affects the movement behaviour of *A. praelongus* at and above this scale. *Acanthophis praelongus* changes its movement pattern in response to wet-dry season cycles [32], as do many other reptile species in Australia’s wet-dry tropics [1,6]. In this analysis, we pinpoint the time scale where such seasonal fluctuations affect movement behaviour.

Broader observations of strong seasonality further suggest that at larger time-scales (greater than the 64 days considered here), movement distances are likely to be cyclic rather than fractal. We do not suggest that all significant aspects of an animal’s behaviour can be captured by the 1/f model. Rather, we use this as a general model to analyse the presence of autocorrelation and the considered time scale. Figure 1 shows that the model fits the data well. Our analysis thus supports the notion that longer ecological time series typically exhibit more variance [33]. Also, the good fit of the 1/f model over the whole spectra suggests that autocorrelations may not be removed by down-sampling the data. Other methods of synchrony account for autocorrelation by AR models of residuals [34]. Given that there is no apparent flattening of the periodograms, such an approach would likely require a high order AR model with few degrees of freedom as a result. The phase-based approach, which is not dependent on choice of AR model, may be most appropriate for analysis of synchrony in these adders.

The 1/f model used for analysis of the autocorrelation pattern has been demonstrated to fit well with weather fluctuations [29]. We here show that it is also an appropriate model for snake movement, suggesting that weather conditions may have been a major driver of snake movements. However, because of the lack of synchrony at time scales below three weeks, the impact of weather conditions must have been limited at this level. Instead, the daily movement of *A. praelongus* appears to be largely driven by factors that vary at the individual level, such as a snake’s internal state, its biotic interactions, or microclimatic factors. In agreement with this conclusion, Brown and Shine [1] analyzed activity of water pythons (*Liasis fuscus*), slatey-grey snakes (*S. cucullatus*) and keelbacks (*Tropidonophis mairii*) at a nearby site and found that, after removing seasonal trends, daily weather factors explained very little of the temporal variance in encounter rates. Given that we would expect ectotherms to be highly sensitive to environmental conditions, these are intriguing results. One explanation for the weak effect of environment in these cases might be that conditions are almost always suitable for movement at night in the tropics where low daily and annual variation in temperatures is the rule [1,12]. Similar studies in temperate regions may, thus, yield different conclusions. Alternatively, subtle behavioural modifications may strongly buffer individuals from environmental variation at short time scales [11]. For example, minor adjustments of a snake’s position can dramatically change the amount of incident radiation it absorbs. Large ectotherms are also buffered from environmental temperatures simply by dint of their mass and thermal inertia [12]. Irrespective of the mechanism, our results suggest that the daily displacement distances of our sample of male *A. praelongus* were insensitive to short-term weather changes during the wet season. The broader climate (e.g., the regime of dry-wet season), or seasonally-variable biotic factors (e.g. prey or habitat availability), nonetheless appear to have a substantial impact on the movement ecology of this species*.* We may therefore find a different cut-off for synchrony during the dry season, where climatic conditions place more constraints on snake movement. Given that our data set only consisted of male snakes, it is also possible that different patterns could be found for females.

Another caveat of our analysis is the small sample size. Although the original dataset followed 54 snakes, our requirement for a long period of uninterrupted observations rapidly reduced our useable data to six snakes over a 64-day period. Examination of other radiotelemetry studies made available to us suggest that data incompleteness, or short time intervals, are common issues with such data. Nonetheless, although our dataset represents only six individuals, the DIC based model selection was able to identify a cut-off time scale of synchronicity. Weak data would mask a difference between models; a situation we didn’t encounter here. Furthermore, our sensitivity analysis revealed that the conclusions are insensitive to the choice of hyperprior. Nonetheless, we advocate caution in the interpretation of our results because the sample size only allows us to compare simple models. With a larger data set, it may be possible to include models that identify multiple cut-offs: for example, where synchronies for higher frequencies could be modelled as being lower, but not necessarily exactly zero. Regardless, we can confidently conclude that the method was able to identify timescales of particular interest for synchrony in this data set.

The analysis of colour parameters indicates that snake movement is positively autocorrelated, since the posterior density of all individual *γ*_*n*_ and population level parameters $\overline{\gamma}$ are larger than 0 (Table 2). Hence, if an individual snake moves a long way one day (or week), it is likely to move far the next day (or week) as well. A similar pattern of autocorrelated movement was found in another snake species at a study site adjacent to the one used in the present study [35]. Slatey grey snakes (*Stegonotus cucullatus*) moved over several successive nights, interspersed with sedentary periods lasting several nights. This pattern of positive autocorrelation among movements was attributed to bouts of foraging interspersed with periods of inactivity to allow digestion [35]. Such patterns may be common among snakes, which typically ingest relatively large meals, but has also been demonstrated for other ectoterms such as cane toads [36] and blue-tongue skinks [37] tracked in the same area.

## Conclusion

Our analysis illustrates the value of spectral analysis in examining movement patterns. When combined with the hierarchical Bayesian framework, we show that the analysis is powerful enough to identify important time scales even with data on only six individuals. By discovering the lack of population-level synchrony at shorter time scales, we obviate the need to check for correlations between daily movement rates and a very long list of potential environmental drivers. We also show, through autocorrelation analysis, that movement is primarily explained by individual history, resulting in autocorrelation over long time periods. Analyzing movement data using spectral analysis will become increasingly important as new technologies (e.g. GPS and satellite tracking) generate large quantities of continuous and highly resolved movement data. We suggest that, for such data, a first critical step will be to determine the autocorrelation structure, and then, where multiple individuals are tracked, the time scale at which synchrony becomes apparent. Exploratory analysis of this kind will rapidly narrow down the possible list of global drivers of movement and the time scales at which they come into effect. This will enable us to make sensible inference about these drivers, their influence, and how they might change in the future. Indeed, studying synchrony can provide insight into the drivers of fluctuations in many ecological systems [19,38]. Here we show that it is important to consider not only the presence of synchrony, but also the timescale at which it comes into effect.

### Availability of supporting data

The data set supporting the results of this article is included within the article (and its additional file).

The results of the sensitivity analysis is also available as an additional file.

## Additional files

Additional file 1: Table S1. Relocation distances. Additional file 2: Figure S1. Sensitivity analysis.

## Abbreviations

WCD: Wrapped cauchy distribution
DIC: Deviance information criterion
MCMC: Markov chain Monte Carlo
AR: Autoregressive
