# Supplementary material for: Identifying the time scale of synchronous movement: a study on tropical snakes
Source: Mov Ecol. 2015 May 4;3(1):12. doi: 10.1186/s40462-015-0038-5 (PMC4418100; doi:10.1186/s40462-015-0038-5)
Supplement: Additional file 2: Figure S1. — Sensitivity analysis. [file 40462_2015_38_MOESM2_ESM.pdf]

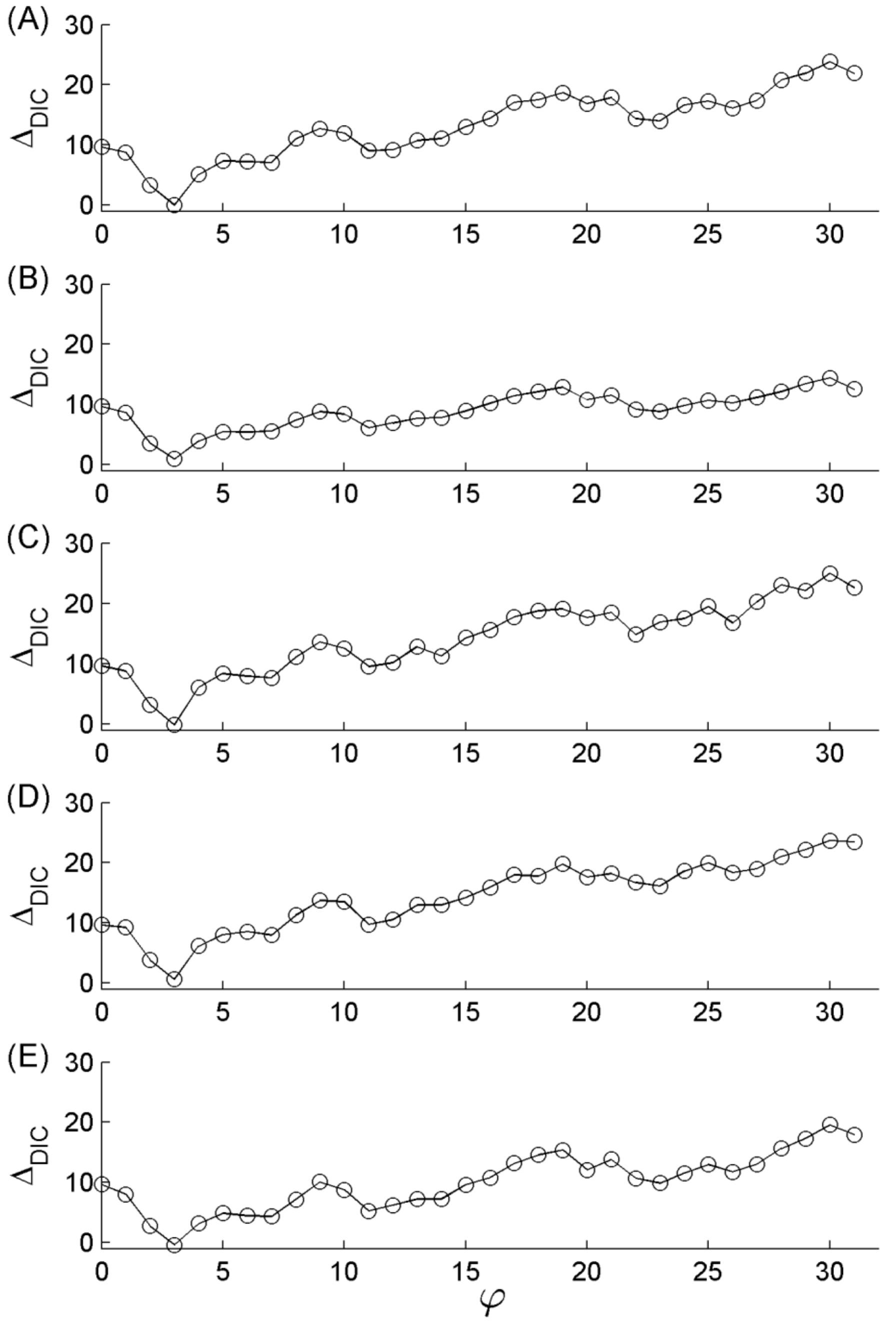

Supplementary figure. The figure shows the differences in DIC (Δ_{DIC}) for models describing the data as synchronous for frequencies $f \le \varphi$ under different hyperpriors. Panel (A) shows the behavior under the elicited prior and contains the same information as Fig. 2 of the manuscript, replotted here to promote comparison. Panels (B) and (C) implement alternative hyperpriors for ν, using a gamma distribution with 95% density within the range 5 to 10 and 100 to 200, respectively. Panels (D) and (E) implement alternative hyperpriors for μ, using Beta(0.5,0.5) and Beta(5,5), respectively. To promote comparison, all Δ_{DIC} values are given relative to the best model under the elicited hyperpriors, i.e. $\varphi = 3$ in panel (A).

The lowest DIC was consistently found for φ=3, with the model with φ=4 having considerably less support than the preferred model (Δ_{DIC} within the range three to seven). This shows that the conclusions are insensitive to the choice of hyperprior.
